# Supplementary material for: Towards integrated tunable all-silicon free-electron light sources
Source: Nat Commun. 2019 Jul 18;10:3176. doi: 10.1038/s41467-019-11070-7 (PMC6639370; doi:10.1038/s41467-019-11070-7)
Supplement: Supplementary file 1 — Supplementary Information [file 41467_2019_11070_MOESM1_ESM.pdf]

Supplementary Information for

Towards integrated tunable all-silicon free-electron light sources

Roques-Carmes et al.

Contents

Supplementary Note 1: Discussion on efficiency and comparison with other technologies 3

Supplementary Note 2: Simulation setup 5

Supplementary Note 3: Experimental setup 9

Wavelength calibration 9

Polarization selection and background definition 9

Identification of the effect responsible for the emission 11

Calibration measurement 11

Supplementary Note 4: Evaluating and matching power estimates from experimental and simulation setups 14

Supplementary Note 5: Additional measurements of aluminum-coated silicon gratings 17

Supplementary Note 6: Design proposition of an integrated Smith-Purcell source 19

Supplementary Note 7: Maximum power estimates 23

Supplementary Note 8: Material factor analysis 24

Supplementary Note 9: Discussion on CMOS-compatibility of our proposed device 26

Nanofabrication of the field emitter and grating 26

Vacuum packaging 26

High voltage packaging 27

Supplementary Note 10: Beam diameter and divergence characterization 27

Supplementary Note 11: Discussion on efficiencies reported in our experiments 28

Supplementary References 31

Supplementary References 31

Supplementary Note 1: Discussion on efficiency and comparison with other technologies

In this section, we give definitions of the efficiency reported in our work and discuss the performance of our Smith-Purcell (SP) near-infrared source in terms of efficiency and tunability. We first define the (optical) power efficiency as the ratio of measured output power to the power of the electron beam

$$\eta_{\text{opt}} = \frac{P_{\text{opt}}}{P_{\text{in}}} = \frac{P_{\text{opt}}}{I \times V}, \tag{1}$$

where I is the electron beam current and V the acceleration voltage. In our experiments, the measured power conversion efficiency is low (see Figure **1**, in the range 6×10^{-8} (at 2 keV) – 3×10^{-7} (at 20 keV) for all-silicon nanogratings).

Another conventional metric to determine the efficiency of light emitting devices is the (external) quantum efficiency, i.e. the ratio of number of output photons to the number of input electrons, which we can also express as a function of the output optical power:

$$\eta_{\text{QE}} = \frac{N_{\text{photons}}}{N_{\text{electrons}}} = \frac{e\lambda_0}{hc}\frac{P_{\text{opt}}}{I}, \tag{2}$$

where λ_0 is the center radiation wavelength, e the electron charge, h is Planck's constant and c the velocity of light in vacuum. We plot the quantum efficiency of a variety of silicon-based light emitting devices as a function of the output wavelength in the near-infrared. In terms of quantum efficiency, our experimental proof-of-concept reaches efficiencies comparable with silicon LEDs and rare-earth doped silicon-based hosts. It is also worth noting that:

- ▷ The output wavelength of our device is continuously tunable, while tunability in other technologies is achieved by design optimization (thus, a single device will output a single center radiation wavelength, which can be tuned by optimizing the device design).
- ▷ In our device, the electron energy is much larger than the photon energy, by 3 to 4 orders of magnitude.
- ▷ The quantum efficiency of our device is one order of magnitude below the continuous-wave Raman laser (reported in [1] – requires optical pumping around 1550 nm) and III-V technology grown on silicon substrates (reported in [2]). We discuss ways of increasing the power efficiency (and subsequently, the quantum efficiency) of our device by several orders of magnitude in Section VII.

We also plot the experimental power and quantum efficiencies of our types of grating (silicon only and aluminum-coated silicon with periods 139 and 278 nm), shown in Supplementary Figure **2**.

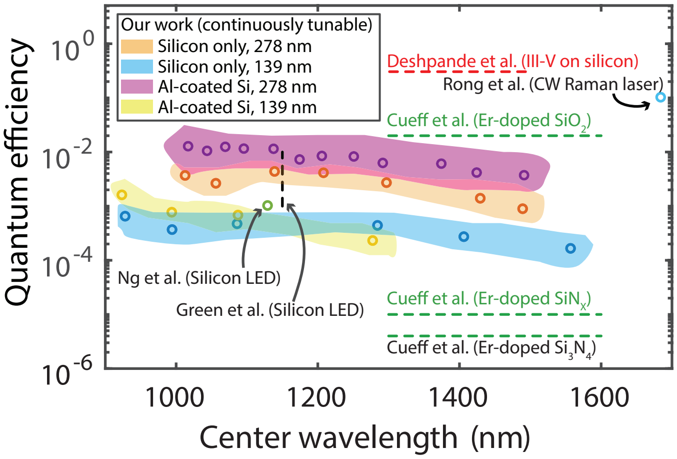

Supplementary Figure 1: **Quantum efficiency comparison between various technologies, including our work.** Dashed lines represent ranges of experimental data from other technologies reported or extracted from references [1–5].

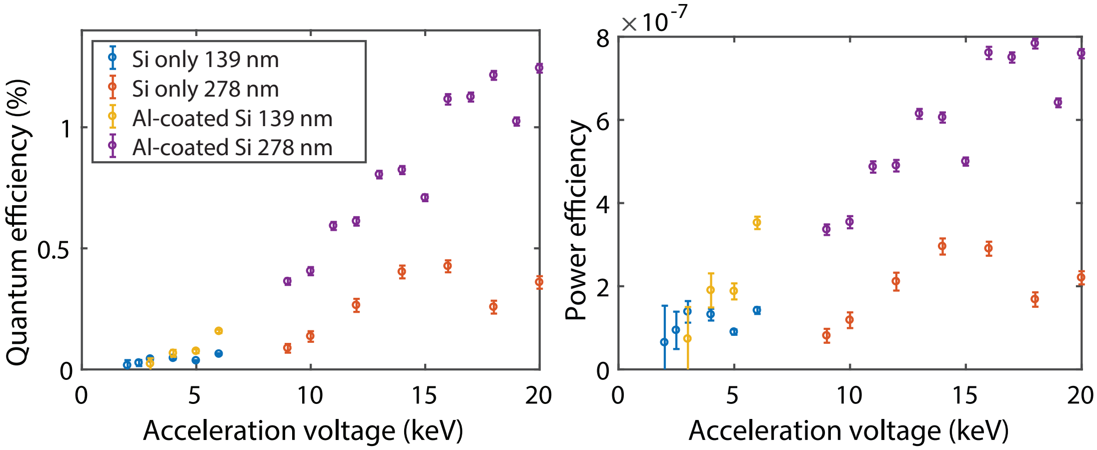

Supplementary Figure 2: **Experimental power and quantum efficiencies. Left:** Quantum efficiency versus acceleration voltage. **Right:** Power efficiency versus acceleration voltage. Error bars are estimated from the noise standard deviation and fitting the signal to a Gaussian.

Supplementary Note 2: Simulation setup

We perform time-domain simulations to estimate the power spectrum of photons emitted by electrons propagating at a given height h above a periodic structure. We design this simulation setup in order to mimic our experiment. With some necessary approximations that we will discuss in the next sections, we can then fit the simulated power spectrum to the measured one with a single fitting parameter.

In our simulation setup, a single electron of charge $-e$ is flying above a grating (here, made of crystalline silicon) at height h and normalized velocity $\beta = v/c$. The grating is periodic of period L along the $\hat{x}$ direction. The trajectory of the electron is in a plane parallel to the grating plane and along its periodicity:

$$\boldsymbol{v}(t) = (\beta c, 0, 0)^T \tag{3}$$

$$\boldsymbol{r}(t) = (x_0 + \beta c\, t, y_0, z_0)^T \tag{4}$$

A commercial-grade simulator based on the finite-difference time-domain method was used to perform the calculations [6]. In time-domain, a convenient way to mimic the polarization field of a moving charged particle is to use a delayed array of dipoles. The current and polarization induced by the trajectory of the electron is given by

$$\boldsymbol{J}(\boldsymbol{r}, \omega) = -e\, e^{-i\frac{\omega x}{v}} \delta(y - y_0)\delta(z - z_0)\hat{x} \tag{5}$$

$$\boldsymbol{P}(\boldsymbol{r}, \omega) = i\frac{e}{\omega} e^{-i\frac{\omega x}{v}} \delta(y - y_0)\delta(z - z_0)\hat{x} \tag{6}$$

The polarization distribution is a continuous function that can be approximated over a finite length (for instance, one unit cell) by a series of dipoles uniformly spaced and delayed in the time-domain. We set their dipole moment to $p_0 = \frac{e\,\mathrm{sim}_x}{N_{\mathrm{dip}}}\delta(z - z_0)$ so that they create a polarization density in the frequency-domain of the form:

$$\boldsymbol{P}_k(\boldsymbol{r}, \omega) = i\frac{e\,\mathrm{sim}_x}{\omega N_{\mathrm{dip}}} e^{-i\frac{\omega x_k}{v}} \delta(x - x_k)\delta(y - y_0)\delta(z - z_0)\hat{x} \tag{7}$$

$$= i\frac{e}{\omega}\,\Delta x\, e^{-i\frac{\omega x_k}{v}} \delta(x - x_k)\delta(y - y_0)\delta(z - z_0)\hat{x} \tag{8}$$

where $\Delta x = \mathrm{sim}_x / N_{\mathrm{dip}}$ is the uniform spacing between dipoles along the $\hat{x}$ direction, equal to the ratio of the simulation length along x to the total number of dipoles N_{dip}. This discretization allows us to approximate the exact polarization density $\boldsymbol{P}(\boldsymbol{r}, \omega)$ as a Riemann sum. We define the

function $\gamma_x : x' \to \delta(x-x')e^{-i\frac{\omega x'}{v}}$.

$$\begin{aligned}
\sum_{k=1}^{N_{\text{dip}}} \boldsymbol{P}_k(\boldsymbol{r},\omega) &= i\frac{e}{\omega}\delta(y-y_0)\delta(z-z_0)\hat{x}\,\Delta x \left(\sum_{k=1}^{N_{\text{dip}}} e^{-i\frac{\omega x_k}{v}}\delta(x-x_k)\right) && (9)\\
&= i\frac{e}{\omega}\delta(y-y_0)\delta(z-z_0)\hat{x}\,\Delta x \left(\sum_{k=1}^{N_{\text{dip}}} \gamma_x(x_k)\right) && (10)\\
&\longrightarrow_{N_{\text{dip}}\to\infty} i\frac{e}{\omega}\delta(y-y_0)\delta(z-z_0)\hat{x}\int \gamma_x(x')\,dx' && (11)\\
&= i\frac{e}{\omega}\delta(y-y_0)\delta(z-z_0)\hat{x}\int \delta(x-x')e^{-i\frac{\omega x'}{v}}dx' && (12)\\
&= \boldsymbol{P}(\boldsymbol{r},\omega) && (13)
\end{aligned}$$

A delayed dipole source in FDTD Lumerical has the following form in frequency-domain :

$$p_{k,\text{FDTD}}(\boldsymbol{r},\omega) = p_{\text{base}}\; s(\omega)e^{-i\frac{\omega x_k}{v}} \tag{14}$$

where p_{base} is the base amplitude imposed by the simulation and $s(\omega)$ is the spectral source norm of the dipole. To match this expression with $p_k(\omega,\boldsymbol{r})$, we must multiply the dipole moment by a normalization factor α defined as:

$$\alpha(\omega) = \frac{e\,\Delta x}{p_{\text{base}}\; s(\omega)\;\omega} \tag{15}$$

This normalization, because of its frequency-dependence, has a dramatic importance in (1) determining the right spectral lineshape and (2) converting our numerical result in “real” units (number of photons, Watts, etc.).

A more comprehensive numerical approach could be performed by taking into account the electron beam diameter and angular spread, in addition to the angle between the electron beam propagation direction and the grating, before integrating over multiple angles and heights of interaction. However, there are two fundamental issues of a simulation of an electron impinging on the surface of a bulk; and how we by-pass them by assuming the electron flies at a constant height h in our simulation setup:

- ▷ If the trajectory of the electron is oblique, some of the dipoles generating the induced polarization will be arbitrarily close to the surface or inside the bulk. This is a problem as soon as the imaginary part of the refractive index is not exactly zero, because of the divergence of the LDOS [7, 8].

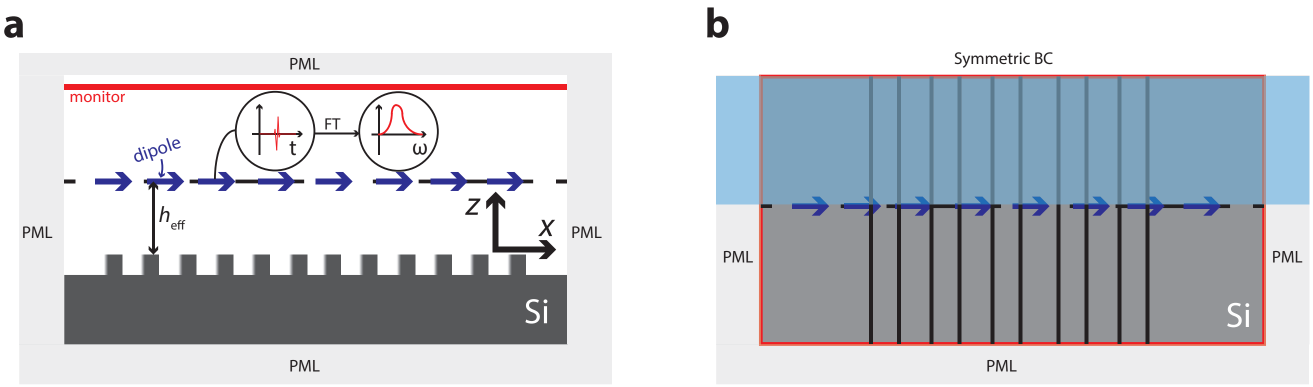

Supplementary Figure 3: **Schematic of the simulation setup.**

▷ Nonlocal effects becomes nonegligible when electrons are very close to the grating ($\leq 5-10$ nm). Such an effect is neglected in our calculation.

Thus, assuming that our electrons fly at a constant height above the grating, we by-pass the two difficulties mentioned above. We chose the height to be equal to the exponential decay factor of fields outside of the grating [9, 10], which does not depend on the material permittivity:

$$h_{\text{eff}} = \frac{\gamma\beta\lambda}{4\pi} \approx \frac{L}{4\pi} \quad \text{in the non-relativistic case} \tag{16}$$

where $\gamma = (1-\beta^2)^{-\frac{1}{2}}$ and λ is the radiation wavelength. To simplify Equation (16), we have used the SP relation, connecting the radiation wavelength to the velocity of the electron and the emission angle θ, measured with respect to the direction of the electron trajectory (forward).

$$\lambda = L\,(\frac{1}{\beta} - \cos\theta) \tag{17}$$

The general form of Equation (17) displays a diffractive integer index m [11]. As we only observe first-order SP radiation in our setup over the wavelength range 800 – 1600 nm, we set $m = 1$.

For the samples studied in this paper, we get $h_{\text{eff}} = 22.75 \pm 0.16$ nm (for $L = 278$ nm) and $h_{\text{eff}} = 11.42 \pm 0.03$ nm (for $L = 139$ nm).

After running the simulation, we can extract the farfield radiation $E(f)$ with a near-to-farfield transformation from a monitor positioned far enough from the electron beam (farther than $\sim 2\lambda$), in units of W/Hz^2 = J/Hz ("nonorm" units of FDTD Lumerical Solutions [6]). Thus, $E(f)$ is analogous to the spectral energy density. The generated number of photons per electron can

readily be derived from the spectral density of energy:

$$N_{\text{SP}} = \left| \int df \frac{E(f)}{hf} \right| \tag{18}$$

The absolute output power can also be computed:

$$\text{P}_{\text{SP}} = \frac{I}{e} \left| \int E(f) df \right| = \frac{I\ c}{e} \left| \int E(\lambda) \frac{d\lambda}{\lambda^2} \right| \tag{19}$$

where I is the incident beam current (for instance, measured from the experimental setup).

Supplementary Note 3: Experimental setup

Here, we give more details on the experimental setup, in complement to Figure 3 from the main text and the section "Experimental setup" in the Methods Section. The Scanning Electron Microscope (SEM) used in this setup is operated in conditions that are very far from conventional imaging conditions (the electron beam current in our experiment varies between 150 nA – 2 μA). In addition, because the electron beam is focused on the sample at a grazing angle, a significant portion of the beam impinges on the sample, thus also producing light from processes that do not abide by the same physics as the SP effect: the output intensity does not scale with the number of unit cells the electron interacts with (among which, transition radiation [12] and incoherent cathodoluminescence [7, 13]). Also, incoherent cathodoluminescence is expected to be very weak in silicon, as it relies on electron-hole radiative recombination (which is superseded by non-radiative recombination processes in silicon, such as Auger recombination [14]). These other sources of radiation are subtracted from the output signal with a polarization-selective measurement (see section below).

Wavelength calibration

The linear InGaAs photodiode array is first calibrated at a central wavelength of 1000 nm (with a wavelength window of ± 200 nm), by using the grating second order from narrow bandwidth lasers at $532 \cdot 2$ nm and $(635 \pm 5) \cdot 2$ nm. We notice that these laser wavelengths are shifted from their calibrated value when moving the central wavelength. Thus, we measure their shifted wavelength as a function of the central wavelength and correct the data with a linear interpolation:

$$\lambda^r = \frac{\lambda_2^r - \lambda_1^r}{\lambda_2^m - \lambda_1^m}\lambda^m + \frac{\lambda_1^m \lambda_2^r + \lambda_1^r \lambda_2^m}{\lambda_2^m - \lambda_1^m} \quad (20)$$

where the λ^r is the real wavelength, λ^m the measured one, and the $\{1, 2\}$ indices denote the two laser peaks used for wavelength calibration.

Polarization selection and background definition

Since its original observation, SP radiation has been shown to be linearly polarized along the electron beam propagation direction [11]. We experimentally verify this observation in Figure 3(b) of the main text, as the minimum of the optical signal is recorded at a polarization perpendicular to

the beam propagation direction. We can utilize this observation in order to subtract the background signal (defined as any other source of electron-beam driven radiation – coming from local defects, coherent and incoherent processes that do not arise from the periodic structure of the material – commonly refered to as cathodoluminescence [7]).

We here assume that radiation from these other processes is polarization-independent. We did not mention other sources of background, that are recorded even when the electron beam does not impinge on the grating (e.g. ambient light): these other sources of background can be counted in the subtracted cathodoluminescence contribution as they are also unpolarized. Using this assumption to define the orthogonal polarization measurement as background translates into the following approximation: $I_{\mathrm{CL}}^{X} - I_{\mathrm{CL}}^{Y} \approx 0$ (where I_{CL}^{α} is the incoherent cathodoluminescence intensity polarized along direction α). We can evaluate the percentage of error introduced in our measurement by making this necessary assumption:

In order to quantify the error induced by making the assumption that the incoherent CL background is polarization-independent, we further analyzed data where the incoherent CL is apparent (139 nm silicon-only grating, 6 keV excitation). Our analysis is shown in Supplementary Figure **4**. After background subtraction, the remaining background level around the incoherent CL peak (corresponding to silicons bandgap) is on the order of 500 counts. This results in a potential overestimation of the number of counts corresponding to the SP peak by 20% for peak wavelengths around 1.1 eV (i.e. 14, 16 and 18 keV for the 278 nm gratings and 5 and 4 keV for the 139 nm gratings). We speculate that this is the main effect responsible for the breaking of the general trend exhibiting lower SP efficiencies for lower electron kinetic energies. This overestimation is absent from the other data points as we fit our signal to a Gaussian-peak in order to estimate the power efficiency.

This approximation is necessary to extract absolute power estimates from our samples (off-the-shelf large area patterned silicon gratings); however, to avoid making this assumption, one could imagine patterning smaller areas and measuring the background signal from unpatterned areas also enclosed in the objectives collection area, which would eliminate the need of a polarization-dependent measurement.

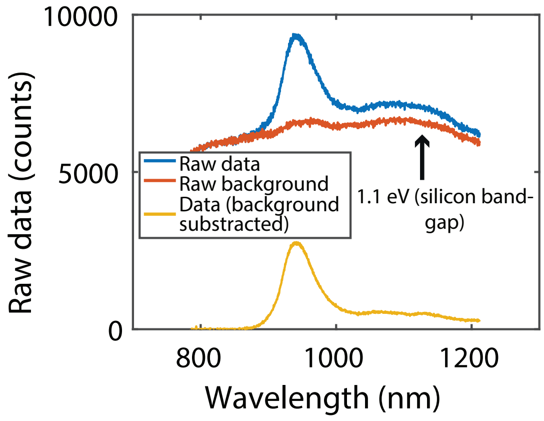

Supplementary Figure 4: **Polarization-dependence of incoherent cathodoluminescence.** The raw data (blue) and background spectra (red) are shown prior to subtracting the background to the raw data (yellow). The incoherent CL peak is apparent around 1.1 eV.

Identification of the effect responsible for the emission

One possible source of concern here is that electrons incident on the silicon grating itself at a glancing angle might produce a modulated cathodoluminescence that may share some of the features of the observed emission radiation. In either case, an interesting and potentially useful on-chip optical source results. However, there are a number of reasons to be confident that the signal primarily results from electrons in an aloof excitation configuration. These are: (1) the electron energy-photon wavelength dependence and its match with the SP theory; (2) the measured bandwidth and its narrowing for low kinetic energies; (3) the reduced efficiency for lower kinetic energies (for which the shorter electron nearfield does not interact as efficiently with the nanograting); (4) the measured polarization of the emission, along the direction of the electron propagation.

Calibration measurement

We are able to measure the absolute value of the SP radiation by performing a calibration measurement described in Supplementary Supplementary Figure (**5**). In our optical setup, the absolute value of the radiation (in units of W/nm) is attenuated by a wavelength-dependent loss function $L(\lambda)$ (which encompasses absorption, reflection, the quantum efficiency of the detector,

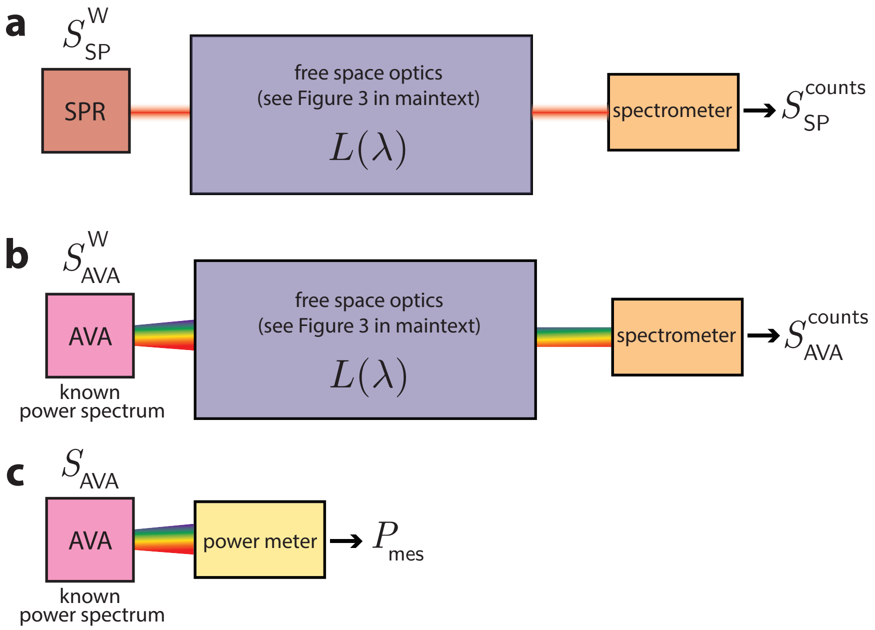

Supplementary Figure 5: **Calibration measurement process.** The SP radiation signal is measured (**a**) through a set of optical components resulting in wavelength-dependent loss function $L(\lambda)$ and detection by the spectrometer of the signal $S^{\text{counts}}_{\text{SP}}$. The known spectrum of the calibrated source AVA is measured (**b**) through the same set of optical components, resulting in the detection by the spectrometer of the signal $S^{\text{counts}}_{\text{AVA}}$. (**c**) The effective area factor is normalized by measuring the integrated power of the calibrated source (AVA) with a power meter.

etc.). The signal detected by the spectrometer is in units of counts/nm, thus $L(\lambda)$ is in units of counts/W. We thus have:

$$S^{\text{counts}}_{\text{SP}} = L(\lambda) \cdot S^{\text{W}}_{\text{SP}} \tag{21}$$

$$S^{\text{counts}}_{\text{AVA}} = L(\lambda) \cdot S^{\text{W}}_{\text{AVA}} \tag{22}$$

where $S^{\text{counts}}_{\text{SP}}$ is the SP signal recorded by the spectrometer (in counts) and $S^{\text{counts}}_{\text{AVA}}$ is the signal recorded from the calibrated source (in counts).

We can get the value of the signal before the losses, knowing the absolute value of a calibrated source and its resulting spectrum as measured by the spectrometer (Supplementary Figure (**5**b)):

$$S^{\text{W}}_{\text{SP}} = S^{\text{counts}}_{\text{SP}} \cdot \frac{S^{\text{W}}_{\text{AVA}}}{S^{\text{counts}}_{\text{AVA}}} \tag{23}$$

The power spectrum of the calibrated source is actually given in units of irradiance (Watts/nm/unit area); we model this dependence per unit area by an effective area factor corresponding to the size

of the emitter. We assume this effective area is wavelength-independent, we can thus compute it by measuring the source total power P_{mes} with a power meter (Supplementary Figure (**5**c)):

$$A_{\text{eff}} = \frac{P_{\text{mes}}}{\int_{\lambda_{\text{min}}}^{\lambda_{\text{max}}} S_{\text{AVA}}(\lambda)\, d\lambda} \tag{24}$$

where S_{AVA} is in units of Watts/nm/unit area, and $[\lambda_{\text{min}}; \lambda_{\text{max}}]$ is the power meter detection bandwidth. Then, we can get $S^{\text{W}}_{\text{AVA}}$ from the data:

$$S^{\text{W}}_{\text{AVA}} = S_{\text{AVA}} \cdot A_{\text{eff}} \tag{25}$$

We sometimes observe negative values of the spectral power, after subtraction of the polarization background. When integrating the total power, we first fit the main peak with a single gaussian distribution, in order not to count the previously mentioned negative values.

We also note that the power meter used in this experiment (Newport 918-UV) has a wavelength-dependent responsivity R_λ, which should be taken into account when evaluating the total power. The displayed power by the power meter assumes that all photons have a given wavelength, set to 532 nm in this experiment. Thus, the measured power can be deduced from the displayed power P_{dis} from the following formula:

$$P_{\text{mes}} = \frac{\int_{\lambda_{\text{min}}}^{\lambda_{\text{min}}} R_{532\text{nm}} S_{\text{AVA}}(\lambda) d\lambda}{\int_{\lambda_{\text{min}}}^{\lambda_{\text{min}}} R_\lambda S_{\text{AVA}}(\lambda) d\lambda} P_{\text{dis}} \tag{26}$$

This calibration relies on the following main approximations:

- ▷ The calibration data taken from the manufacturer was still valid when performing the experiment shown in Supplementary Figure (**5**b) (according to the data sheet, it should only be valid within the first 50 hours of use).
- ▷ We assume the calibrated source effective area factor A_{eff} is wavelength-independent.

Supplementary Note 4: Evaluating and matching power estimates from experimental and simulation setups

To fully describe our simulation setup, we need to determine the number of unit cells above which the electron is flying. This number is finite and a function of the angle between the electron beam propagation direction and the horizontal. In our experiment, the electron beam is incident on the sample at a nonzero angle which determines a finite number of unit cells around the point of collision with the surface, in which the electrons interact and generate SP radiation. The radiation contribution from other areas, farther from the point of interaction, is negligible. The backscattering coefficient η that sets the portion of electrons reflected while maintaining their angle of incidence can be generally estimated as [15]:

$$\eta = \frac{1}{(1+\sin\theta)^p} \tag{27}$$

where $p = 9/\sqrt{Z}$ and Z is the material atomic number. In the case of pure silicon and an imping-ing angle of $\sim 1^\circ$, we get $\eta \sim 0.95$, which means that most electrons get elastically scattered. This allows us to derive an analytic relation between θ_{fit} and N_{UC} (see Supplementary Figure **6**(b)).

We use the electron beam angle (that sets the number of unit cells) as the single fitting parameter to match the total power of each simulation to the measured power. The number of unit cells N_{UC} can be equivalently chosen as the single fitting parameter, as N_{UC} and θ_{fit} are connected by a simple geometrical construction shown in Supplementary Figure **6**(b), assuming elastic scattering of the electron. This allows us to determine an effective simulation setup matching our experimental results. First, we determine the effective spectral density of energy per unit cell, by running the simulation for a large number of unit cells N_{UC} (here, 110 unit cells is sufficient). We observe that the number of photons per electron per unit cell, or equivalently the output power per unit cell (for a given electron beam current) converges for large number of unit cells. Equivalently, the total number of photons per electron is a linear function of the number of unit cells in the simulation, as can be seen in Supplementary Figure **6**(a). Supplementary Figure **6**(a) is plotted for $L = 139$ nm and an electron with a kinetic energy of 3 keV at a distance $h_{\text{eff}} = 11.2$ nm from the grating, but we observe similar behaviors with different energies and geometries. For large number of unit cells N_{UC}, we can thus approximate the proportionality factor of this linear dependence as the ratio of the output power to N_{UC} for large N_{UC} (in the case plotted in Supplementary Figure **6**(a), the error is of $\sim 5\%$, which is reasonable as we are just interested in fitting the simulation data to

our experiment.

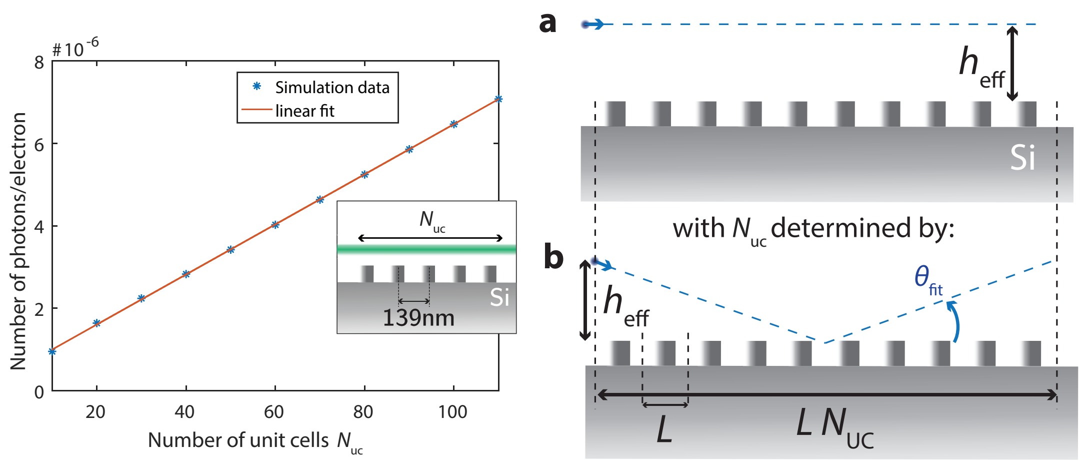

Supplementary Figure 6: **Left: Convergence of the output power per unit cell for large number of unit cells.** The total output power is fitted to a linear function of the number of unit N_{UC} cells with $R > 0.99$. We can thus approximate the linear factor by the ratio of output power to N_{UC} for large N_{UC}. **Right : Determination of fitting angle parameter θ_{fit}.** In our simulation setup (**a**), we assume a single electron flies at the effective height h_{eff}, given by Equation (16), above a finite number of unit cells N_{UC}. N_{UC} is determined by fitting the total power from the simulation to the experimental data. θ_{fit} can be determined from N_{UC} with the geometrical construction shown in (**b**).

We then determine the effective number of unit cells by matching the total power from the simulation to the experimentally measured power. Eventually, we can deduce the effective number the fitting angle θ_{fit} as

$$\theta_{\text{fit}} = 2 \arctan \frac{h_{\text{eff}}}{L\, N_{\text{UC}}} \tag{28}$$

Fitting results are reported for the 139 and 278 nm grating experiments in Table **1**. We get values of the fitting parameters $\theta_{\text{fit}} = (0.0020 \pm 0.00059)^\circ$ for 139 nm and $\theta_{\text{fit}} = (0.0017 \pm 0.00071)^\circ$ for 278 nm pitch gratings. The value of the effective angle is much larger than the angle measured in our experimental setup $(1 \pm 0.5)^\circ$. This may come from the fact that we are neglecting the angular spread of the electron beam and its diameter. However, we get consistent values between the 139 and 278 nm-pitch gratings experimental data, which means our method is consistent.

We observe different lineshapes between the numerical and experimental setups. We speculate that this difference originates from the angular Heaviside window function used in the near-to-

E_i (keV)	Current (nA)	Measured power (pW)	Number of SP photons per unit cell	N_{UC}	θ_{fit} (deg.)
**139nm-period silicon-only gratings data**					
2	161	21	3.90×10^{-8}	4038	0.0023
2.5	243	57	4.39×10^{-8}	5787	0.0016
3	347	140	6.43×10^{-8}	6410	0.0014
4	477	250	8.83×10^{-8}	5371	0.0017
5	889	400	1.20×10^{-7}	3020	0.0031
6	1130	960	1.44×10^{-7}	4343	0.0021
**278nm-period silicon-only gratings data**					
9	1086	790	2.41×10^{-7}	3019	0.0031
10	1508	1780	2.61×10^{-7}	4420	0.0021
12	1120	2840	3.43×10^{-7}	6815	0.0014
14	1236	5110	4.15×10^{-7}	8679	0.0011
16	1424	6610	4.60×10^{-7}	9029	0.0010
18	1495	4520	5.33×10^{-7}	4923	0.0019
20	1685	7420	6.17×10^{-7}	5875	0.0016

Supplementary Table 1: **Data and fitting parameters of experimental and simulated setups.**

farfield transformation in our numerics.

Supplementary Note 5: Additional measurements of aluminum-coated silicon gratings

In this section, we present additional experimental results demonstrating tunable spontaneous emission from aluminium-coated periodic structures. These structures are not made of silicon alone, yet aluminium is the preferred material for metallization in back end of the line very large scale integration (VLSI) processes [16].

We deposited aluminium to a thickness of 10 nm on the grating sample using an electron-beam evaporator system. The electron-beam evaporation was performed at room temperature, with a chamber pressure below 2×10^{-6} Torr and an evaporation rate of 5 Å/s.

We notice that aluminium-coated silicon gratings result in a slightly better SP efficiency, compared to pure silicon samples (defined as the ratio of output power to the input current times the electron beam voltage) which could be due to a better conductivity of the aluminium-coated samples. However, this could be bypassed with silicon-only samples by tuning their extrinsic doping.

These specific samples yield larger efficiencies when excited by fast electrons ($\sim$ 20 keV). These larger efficiencies may originate from (1) larger material factors in aluminum for these wavelength and bandwidth (see Supplementary Figure **10**); (2) larger conductivities in metallic sample resulting in smaller charging and Coulomb repulsion on the surface of the nanograting. Nonetheless, we observe that the efficiency of aluminum samples becomes comparable to the one of bare silicon samples as we reduce the electron energy ($\sim$ 4 keV). This experimental observation hints at the superior material factor – and thus radiation enhancement – of lossless dielectrics, such as silicon, for narrow bandwidth applications (such as in C-band telecommunications).

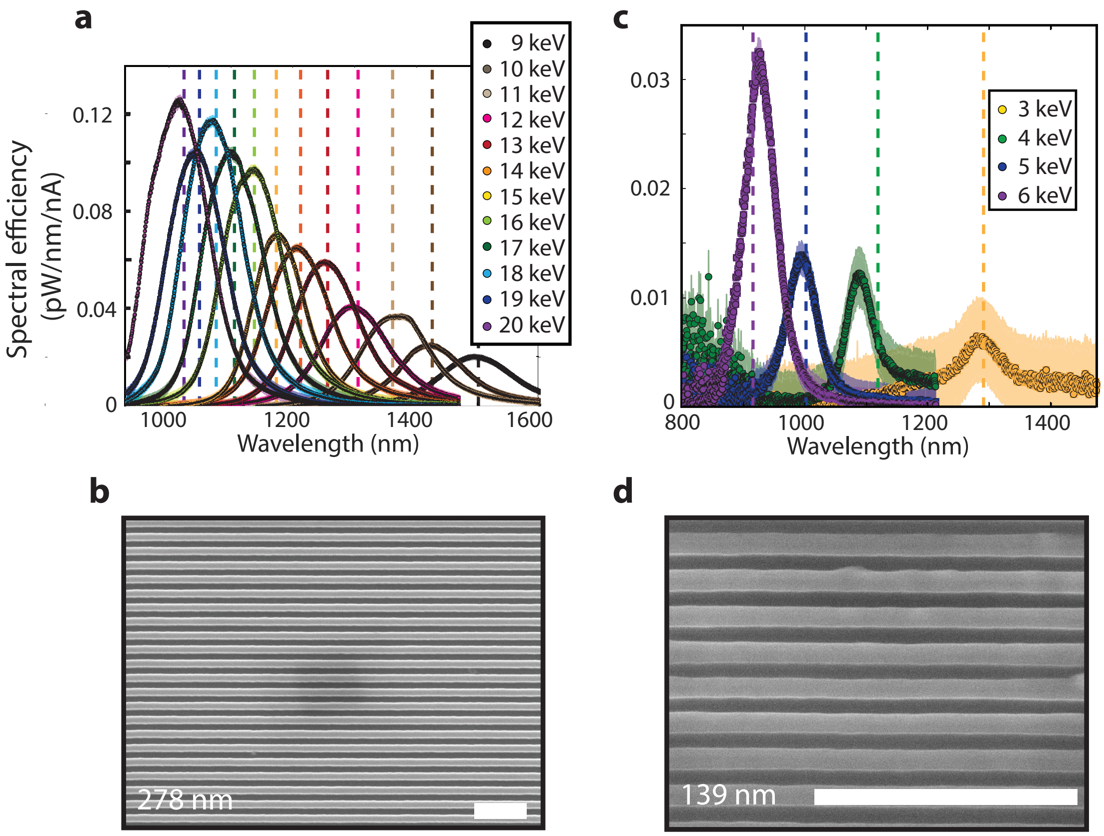

Period	139nm				278nm											
E_i (keV)	3	4	5	6	9	10	11	12	13	14	15	16	17	18	19	20
Current (nA)	166	239	455	489	805	672	633	686	678	608	754	530	635	620	620	625
Measured power (pW)	36	182	427	1034	2434	2379	3390	4032	5416	5157	5653	6454	8098	8744	7552	9493
η_{QE} (%)	0.023	0.067	0.075	0.16	0.36	0.41	0.59	0.61	0.80	0.82	0.71	1.1	1.1	1.2	1.0	1.2
η_{opt} ($\times 10^{-7}$)	0.73	1.9	1.9	3.5	3.4	3.5	4.9	4.9	6.1	6.1	5.0	7.6	7.5	7.8	6.4	7.6

Supplementary Figure 7: **Additional measurements of aluminum-coated silicon gratings.** Experimental measurement of SP spectral efficiency from **(a)** (resp. **(c)**) a 278nm (resp. 139nm)-period aluminium-coated silicon grating. Corresponding scanning electron micrographs are shown in **(b)** (278nm) and (d) (139nm). The white scale bar corresponds to 1μm. The table below summarizes the experimentally recorded data.

Supplementary Note 6: Design proposition of an integrated Smith-Purcell source

In this section, we discuss the integration of a silicon-only SP source with different designs of Field Emitter Arrays (FEA). The fabrication and study of silicon FEA is an active area of research [17]. We list a couple reasons why silicon FEA technologies are attractive today (for a more comprehensive review, we invite the reader to refer to [17]):

- ▷ **Scalability.** The development of silicon FEA has been bolstered by their scalability into VLSI processes, which is superior to their metallic counterparts (Spindt-type FEA).
- ▷ **Density of integration.** Thanks to better fabrication techniques and scalability with silicon-only wafers, silicon FEA are also more promising in terms of density of emitters per unit area. A larger density of emitter results in a larger output current per unit area (flux).
- ▷ **Output power.** One of the reasons why Spindt-type emitters (metallic, usually made of molybdenum) have been prefered was their ability to output larger currents per tip. However, silicon FEA with output currents comparable to Spindt-type FEA have been recently reported [18].
- ▷ **Longevity.** More sophisticated designs have been recently proposed to embed silicon FEA with current limiters into dielectric matrices, thus achieving long lifetimes ($>$ 100 hours) [19].

All these reasons have pushed the community towards ameliorating silicon FEA design in the recent years, in order to achieve better beam characteristics (beam current, diameter, pulsed electron emission, etc.).

Let us describe the basic physical processes of FEA (field emission processes from metals and semiconductors are both based on quantum tunnelling): a sharp nanometer-scale tip (usually conical) is applied a large electric field. This electric field can be a DC field from a gate in the vicinity of the tip (gated FEA), or a pulsed field coming from a powerful laser (pulsed FEA). In both cases, the strong field enables the tunnelling of electrons at the tip of the emitter. After being emitted into vaccuum, these free electrons can be accelerated and focused thanks to a set of adequate cathodes and other gates. The kinetic energy of electrons close to the tip is very small (close to the Fermi Level of the emitter), while cathodes are usually biased at a much larger potential ($>$ 1 keV for most applications and characterization). The cathode is also positioned far

from the tip: thus, the kinetic energy of the electrons in the vicinity of the cathode can be assumed to correspond to the bias voltage of the cathode.

Usually, the addition of focusing gates results in a focusing current of electrons going through this gate, which is lost (these electrons cannot tunnel and do not contribute to the current at the tip): thus, FEA designs with focusing gates usually demonstrate lower currents at the cathode, but smaller beam diameters.

Based on these observations, we can propose a general design for a tunable SP source integrated with a gated FEA, as shown in Supplementary Figure (**8**). A field emitter array is mounted perpendicular to a periodic structure (e.g. a simple grating). This field emitter array can be added several electrodes to facilitate electron tunnelling and achieve better beam focusing. The array is gated at a voltage in the range 10-100 V and the anode is gated a larger voltage of $\sim$ 1 keV. The anode voltage can be tuned in order to accelerate the electrons to a higher energy, thus resulting in a shorter radiation wavelength. The periodic structure is placed at a relatively large distance from the cathode, in order to make sure the electrons fly at a speed which approximately corresponds to the anode voltage.

Thanks to the recent development of reliable, high-output, silicon-only field emitter arrays and the fabrication of small-pitch periodic gratings, the design of an integrated SP source (Supplementary Figure **8**) could be realized with silicon-only structures. This would result in a tunable, compact, silicon-only source, emitting radiation in the near infrared. This design could also be transferred to other wavelength regimes, but other materials may be more efficient at these other wavelengths [10].

We also note that the perspective of integrating SP sources with pulsed field emitter arrays is particularly interesting, as such designs could facilitate the bunching of free-electrons at the radiation wavelength, in order to achieve more efficient emission, and potentially lasing. Such techniques are already used in free-electron lasers [20].

In this work, we compare different designs of silicon [18, 19, 21] and Spindt-type FEA [22, 23] and compute the maximum output optical power of a SP source embedded with these designs as electron sources. To make a fair comparison, we chose designs in the literature that had several points in common:

- ▷ Experimental characterization of the anode current vs. gating voltage is provided, with Fowler-Nordheim coefficients.

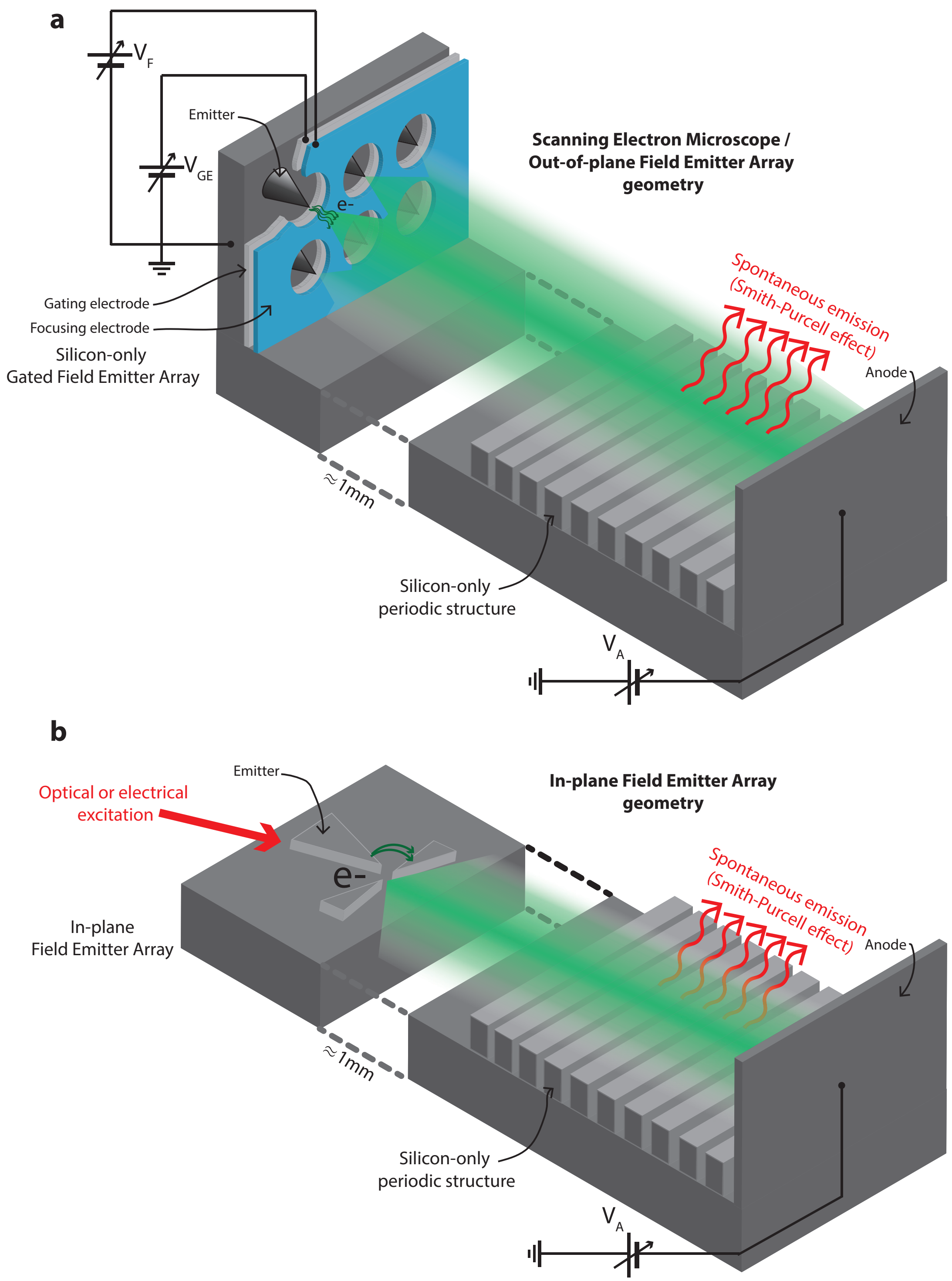

Supplementary Figure 8: **Design proposition of a FEA integrated with a Smith-Purcell source, resulting in a silicon-only integrated tunable source.** (a) SEM/Out-of-plane FEA geometry. (b) In-plane FEA geometry.

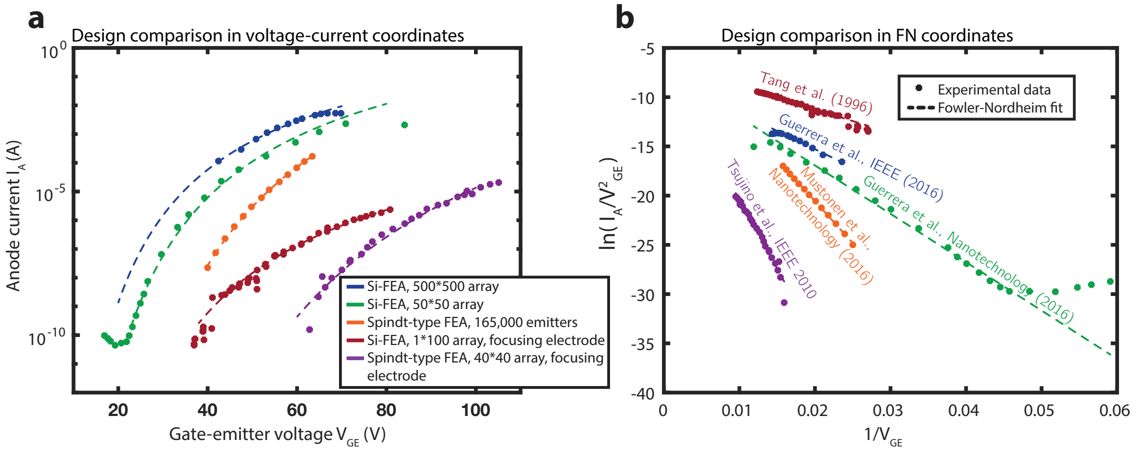

Reference	Number of emitters	$\ln a_{FN}$	b_{FN} (V)	V_0 (V)	V_{sat} (V)
Guerrera et al. [18]	1	-9.39	785	31	60
Guerrera et al. [18]	2,500	-7.1	491.3	22	80
Guerrera et al. [19]	250,000	-7.865	371	20	70
Mustonen et al. [23]	165,000	-3.583	849.21	40	63
Tang et al. [21]	100	-14.89	549.2	38	80
Tsujino et al. [22]	1,600	-6.435	1399	60	100

Supplementary Figure 9: **FEA voltage-current data and Fowler-Nordheim curves taken from references.**

- ▷ The anode voltage is fixed to 1 keV in the characterization experiment. Also, the anode is positioned at a large distance from the cathode ($\gtrsim$ 1 mm). Similar ranges of gating voltage are used (10-100 V). Voltage-current data and Fowler-Nordheim curves taken from these references are shown in Supplementary Figure **9**.
- ▷ For designs where a focusing gate is used, data on the electron beam diameter can be extracted from the paper.
- ▷ For the first three designs [18, 19, 23], similar emitter densities are achieved.

Thus, comparing [18, 19] to [23] allows us to compare state-of-the-art silicon emitter designs to state-of-the-art Spindt-type emitter designs. Comparing [18, 19, 23] to [21, 23] allows us to determine the influence of the focusing gate (via the electron beam diameter parameter) on the SP

radiation output power.

Supplementary Note 7: Maximum power estimates

In this section, we use a theory derived in one of our recent works [10]. In this paper, we derive a universal bound on the photon emission and energy loss of free electrons interacting with arbitrary photonic media; this theoretical bound was shown to yield predictions that matched experimental results [10]. We use this bound to estimate the maximum power and efficiency of a silicon-only integrated SP source based on the different designs from the literature described in the previous section.

It has been shown that for a given electron structure separation d, there exists a shape-independent upper bound on the spontaneous radiation rate of a free electron [10]

$$\frac{d\Gamma(\omega)}{dx} \leq \frac{\alpha}{8\pi c}\frac{|\chi|^2}{\mathrm{Im}\chi}\frac{\psi}{\beta^2}\left[(\kappa_\rho d)K_0(\kappa_\rho d)K_1(\kappa_\rho d)\right]. \tag{29}$$

where α is the fine structure constant, c is the speed of light, χ is the material susceptibility, ψ is the smallest opening angle of a sector (centered at the electron beam) that can enclose a structure, $\kappa_\rho = \omega/c\beta\gamma$ ($\beta = v/c$ and $\gamma = 1/\sqrt{1-\beta^2}$), and d is the separation between electrons and a structure.

Equation (29) imposes a constraint on the maximal radiation that can be obtained from a single frequency. However, for a given desired bandwidth, power-bandwidth limitations come to play [24]. In this perspective, the material factor $\frac{|\chi|^2}{\mathrm{Im}\chi}$ should be replaced by the following expression

$$\mathrm{MF}(\lambda) = \frac{\omega_0}{\Delta\omega}\frac{(\epsilon-1)^2}{\epsilon} = \pi\beta c\left(\frac{1}{\beta^2}-1\right)\frac{(\epsilon-1)^2}{\epsilon} \tag{30}$$

$$\approx \frac{\pi c}{\beta}\frac{(\epsilon-1)^2}{\epsilon} \quad \text{for non-relativistic electrons.} \tag{31}$$

where we have assumed that L, β and λ are connected by Equation (17). Based on Equation (29), we are able to estimate the maximal power P_{max} of a free electron radiation device given its current I, electron beam area $S = \pi D^2/4$, and device length L_G.

$$P \leq P_{\mathrm{max}} = \int_{\omega_{\mathrm{min}}}^{\omega_{\mathrm{max}}} d\omega \int_0^{L_G} dx \int dS \frac{d\Gamma(\omega)}{dx}\frac{\hbar\omega I}{S} \tag{32}$$

$$= \int_{\lambda_{\mathrm{min}}}^{\lambda_{\mathrm{max}}} d\lambda \frac{2\pi c}{\lambda^2}\int_0^{L_G} dx \int dS \frac{d\Gamma(\omega)}{dx}\frac{hc\, I}{\lambda\, S} \tag{33}$$

where the integration bandwidth can be calculated from the Smith–Purcell formula.

Here we assume that the current density is constant over the entire beam area and that the anode current I_A – gating voltage V_{GE} relation is given by the Fowler-Nordheim relation

$$\ln \frac{I_A}{V_{GE}^2} = \ln a_{FN} - \frac{b_{FN}}{V_{GE}} \tag{34}$$

where a_{FN} and b_{FN} are Fowler-Nordheim (FN) coefficients. When they are not given in the references, we extract them from the literature for the five designs we are comparing in this analysis. For each design, we assume this relation is valid only over a range of voltages $[V_0, V_{\text{sat}}]$. A summary of the coefficients, the current - voltage and FN characteristic plots are shown in Supplementary Figure **9**. The FN relation is an empirical equation giving a better fit with experimental data at intermediate gating voltages (for gating voltages large enough so that the field emitter is turned on; but low enough to prevent saturation, as can be seen in the plots of Supplementary Figure **9**).

To summarize results from the main text, power efficiencies of up to $> 10\%$ are reasonably achievable, with careful engineering of the interaction of the electron beam with the nanograting, and phase-matching the electron beam excitation with a resonant mode of the structure. These would translate into quantum efficiencies of up to > 100 (quantum efficiencies larger than 1 are allowed because of the large discrepancy between the electron and photon energies). For these large efficiencies, most of the electron energy would be turned into output radiation, thus significantly modifying the electron trajectory (while, in general, a no-recoil approximation is made because of the large energy mismatch between the electron and the emitted photon). In particular, the significant energy loss from the electron and subsequent red-shift of the outcoming radiation can be corrected by adequately chirping the periodicity of the structure.

Supplementary Note 8: Material factor analysis

The maximum output power of an integrated SP source is proportional to the Material Factor MF(λ). Silicon's material factor is plotted in Supplementary Figure **10**(a) as a function of wavelength for two different grating periods. The influence of the response bandwidth is especially critical for lossless dielectrics such as silicon [24], as can be seen in Supplementary Figure **10**(b): while lossy metals' material factor (such as aluminium and gold) does not really depend on the response bandwidth, silicon's material factor can be greatly increased by resorting to low energy electrons.

In the range of kinetic energies recorded in our experimental setup, aluminium should yield

a larger output optical power, which corresponds to the tendency we observe when comparing results in Figure 2 (main text) and Supplementary Figure **7**, and both materials are CMOS-compatible. For lower kinetic energies (< 30V), silicon material factor beats aluminium's. In the zero-bandwidth limit, silicon's material factor diverges, while aluminium's is limited by its intrinsic loss, as can be seen in Figure 1(b) in Ref. [10]. This knowledge of material's responses can be leveraged to optimize the output of an integrated SP source, for a given bandwidth and target wavelength response.

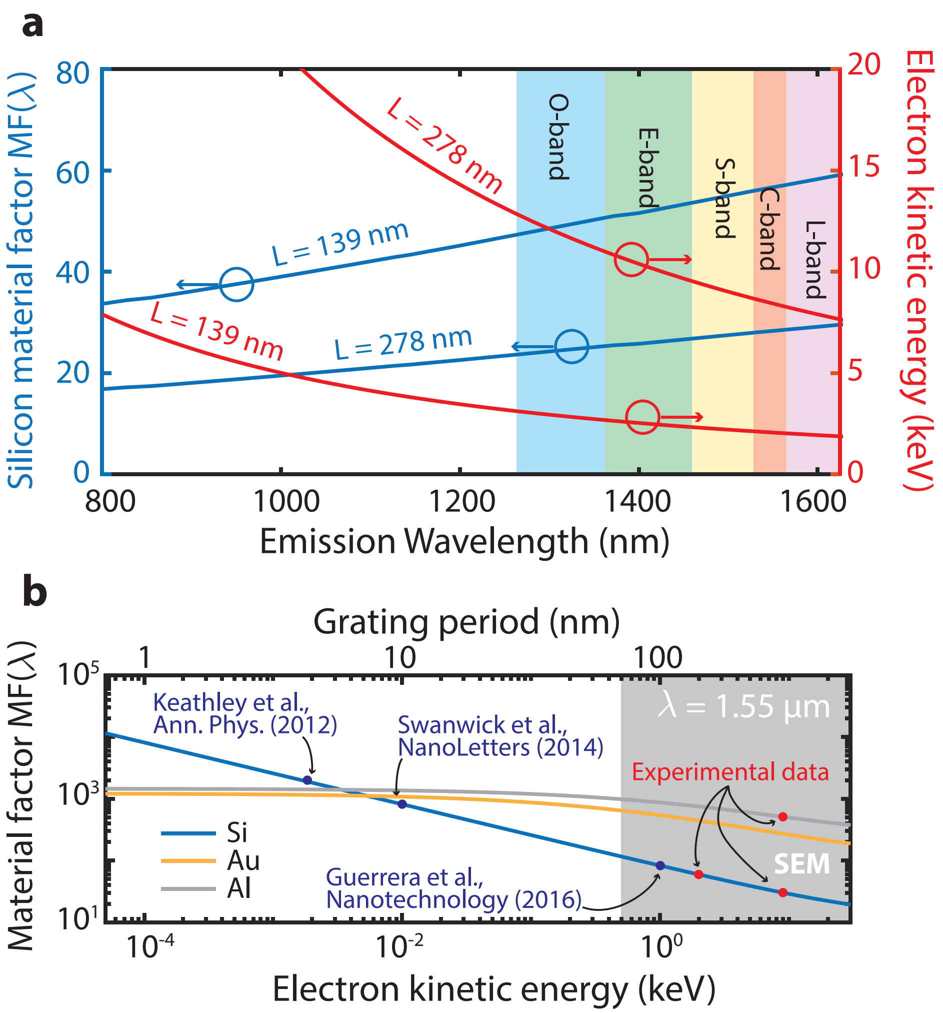

Supplementary Figure 10: **Material factor analysis.** **(a)** Silicon material factor as a function of wavelength (blue) and SP dispersion relation (red). **(b)** Material factor of silicon, gold and aluminium as a function of the electron kinetic energy at $\lambda = 1.55\ \mu$m. The grey shaded area corresponds to kinetic energies usually accessible in a SEM.

	20 keV	1 keV	100 eV	10 eV
**10^{-6} mbar**	10 km	2 km	632 m	200 m
**1.33 mbar**	1.4 cm	2.8 mm	885 μm	280 μm
**13.3 mbar**	1.4 mm	280 μm	88 μm	28 μm
**66.5 mbar**	280 μm	56 μm	18 μm	6 μm

Supplementary Table 2: **Predicted electronic mean free path in air for various pressure and electron energies. Extracted from [15] for a minimum scattering angle of 1 deg.**

Supplementary Note 9: Discussion on CMOS-compatibility of our proposed device

Nanofabrication of the field emitter and grating

The fabrication of the silicon nanograting and in-plane field emitter arrays shown in Figure 1 of the main text is readily CMOS-compatible as these structures are common practice in silicon photonics [25]. Thus, the fabrication of these structures could benefit from the high-yield and low-cost of monolithic integration. The full proposed device is more complex than this simple nanofabrication step, as it requires high-voltage and vacuum operation. We argue below that these two hurdles can be dealt with proper packaging of the silicon dye.

Vacuum packaging

The mean free path of an electron in vaccum at ambient temperature is a function of its energy and of the air pressure [15]. We give its predicted value for various electron kinetic energies and pressures in Table **2**.

In an SEM, the vacuum level is of around 10^{-5} mbar, which corresponds to electronic mean free paths of the order of kilometers, such that less than 10^{-4}% of the beam does not reach the specimen. For our proposed device, the vacuum requirements are much less stringent. Operating at 1 keV, even a vacuum level of 1.33 mbar (= 1 Torr) would result in mean free paths on the order of millimeters. Recently, CMOS-compatible vacuum packaging of MEMS and CMOS chips have been demonstrated for monolithic integration, down to 20 mTorr (= 0.027 mbar) [26, 27]. These packaging techniques would already enable the operation of our device in medium vacuum operation down to at least 10 V.

High voltage packaging

The second potential hurdle for CMOS-compatibility is the required high-voltage operation of our device. However, this hurdle can also be addressed with integrated CMOS high voltage supplies that are commonly developed for lab-on-chip systems [28–30]. They can operate in a DC regime and up to hundreds of volts.

Supplementary Note 10: Beam diameter and divergence characterization

In this section, we describe additional measurements realized in order to characterize the SEM beam characteristics and the discrepancies with the idealized scenario described in Figure 4 of the main text. In order to measure the beam diameter, we assume a Gaussian profile of its current density. We measure the secondary electron intensity (SEI-REF) signal along a line perpendicular to a sharp edge (here, a conductive razor blade). The measured signal can be fitted to an error function (convolution of the Gaussian beam profile with perfect step function). We performed the measurement in similar conditions that those of Figure 2 in the main text (current is maximized at a given acceleration voltage). Results are shown in Supplementary Figure **11** (Left), where we added an empirical fit to our data. We summarize below the assumptions of this fit:

In regular operation conditions, when neglecting chromatic aberrations and aperture diffraction, the minimal beam diameter of an SEM can be expressed as [31, 32]

$$D_{\text{min}} = \left(\frac{4}{3}\right)^{3/8} \left(C_0^3 C_S\right)^{1/4}, \tag{35}$$

where $C_0 = \sqrt{\frac{4I}{b\pi^2}}$ and C_S is the spherical aberration coefficient ($C_S \approx 300$ mm for a working distance of 28 mm). In the following, we identify the beam diameter to the Full Width at Half Maximum (FWHM) of the fitted Gaussian profile. The SEM brightness b scales linearly with the acceleration voltage E

$$b = D_b E. \tag{36}$$

Fitting our data to this model, we get $D_b \approx 5.2 \times 10^3$ A/m^2/sr/eV. This yields a brightness at 20 keV of b(20 keV) $\approx 10^3$ A/cm^2/sr, which corresponds to conventional values of Tungsten emitters [31, 32].

Additionally, we can measure the beam standard deviation as a function of the distance to focus, by mechanically shifting the position of the sample while maintaining a constant working

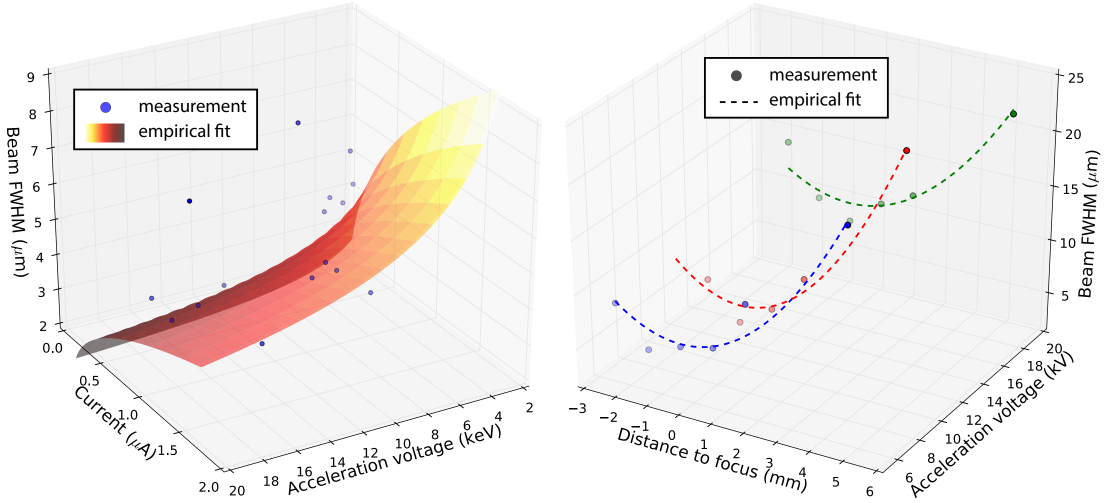

Supplementary Figure 11: **Beam characterization. Left:** Measurement of beam standard deviation as a function of beam current and acceleration voltage. Our measurements are fitted to empirical models of the electron beam [31, 32]. The two outlier points at 10 and 20 keV (for which a large bias voltage is allowed by the SEM software) were excluded from the empirical fit. **Right:** Characterization of beam divergence: measurement of beam standard deviation as a function of acceleration voltage and distance to focus. Our measurements are fitted to a quadratic (Gaussian-beam-like) divergence.

distance. Based on wave-optical models of electron-probe formation in SEMs [31], we fit the beam divergence to a quadratic (Gaussian-like) model

$$D(\Delta f) = D_0 \left(1 + \left(\frac{\Delta f}{\delta}\right)^2\right), \tag{37}$$

where D_0 is the minimal beam diameter (measured at focus) and δ is the longitudinal beam divergence coefficient. Fitting our data to this model in Supplementary Figure **11** (Right), we get divergence coefficients $\delta = 3.65, 3.21$ and 4.14 mm for 5, 10 and 20 keV. The large value of these coefficients compared to our collection characteristic length ($\lesssim 1$ mm) shows that we can safely neglect divergence in our model of the SEM experiment.

Supplementary Note 11: Discussion on efficiencies reported in our experiments

In this section, we quantitatively describe different factors resulting in the discrepancy between the maximal theoretical efficiency showed in Figure 4 of the main text, and our experimental results.

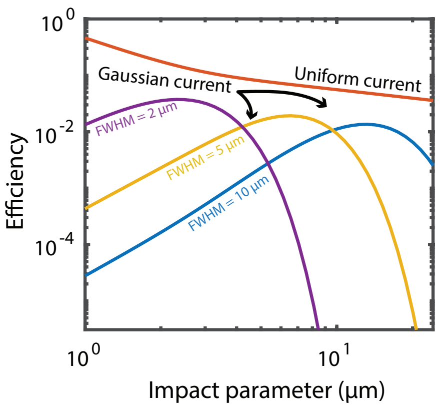

Supplementary Figure 12: **Influence of the beam current distribution on the power efficiency upper bound (**$L = 500\ \mu$**m).** Maximal efficiency versus impact parameter (measured from the center of the beam). For the uniform distribution, the radius is taken to be equal to the impact parameter (optimal configuration).

We believe that the main discrepancy between our theoretical model in Figure 4 and our experiment arises from the electron beam shape and current distribution. Our experimental characterization of the electron beam suggests a Gaussian-like profile of the current distribution, while we have assumed a uniform current distribution in Figure 4 for the sake of simplicity. The upper efficiency for various parameters is shown in Supplementary Figure **12**. For current distributions with bounded circular domains of definition, the optimal impact geometry is when the electron beam is tangential to the grating. However, in the case where the distribution domain is unbounded, the upper efficiency diverges (but could be regularized by taking into account non-local effects [10]). We here regularize it by forcing the distribution domain to be circular, giving the following current distribution with total current I_0:

$$I(y,z) = \frac{I_0}{\left(\mathrm{erf}\left(\frac{R}{\sqrt{2\sigma^2}}\right)\right)^2} \frac{\mathbb{1}\{t^2 \le R^2\}}{2\pi\sigma^2} \exp\left(-\frac{t^2}{2\sigma^2}\right), \tag{38}$$

$$t^2 = y^2 + (z-R)^2, \tag{39}$$

where $\sigma = \mathrm{FWHM}/(2\sqrt{2\log 2})$ is the beam standard deviation, and R its impact parameter. For the Gaussian current distribution of a given FWHM, there is always an optimal impact parameter maximizing the efficiency. Even when optimized, the power efficiency of the Gaussian beam is roughly one order of magnitude below the efficiency of the uniform beam with similar impact parameter.

Additionally, in our model in Figure 4 of the main text, we use a shape-independent form of

the general bound from Ref. [10], which clearly exhibits the dependence on the impact parameter of the electron beam. This shape-independent bound is off from the exact bound by roughly a factor of 5. To achieve efficiencies comparable to the bound, one need to phase-match the electron beam excitation with a resonance of the photonic structure [8]. In our case, this enhancement of the efficiency is bandwidth-limited [24]. Because no particular engineering of the photonic band structure was performed in our experiment, we expect to also be off by a factor comparable to the material factor MF(λ) which we introduce in the main text.

In our experiment, we are only collecting a finite portion of the photons generated by the electron-beam in the solid angle 4π. Because our collection area (≈ 1 mm^2) is small compared to the objective radius, we can assume photons are generated from a point source. Making the extra assumption of an isotropic source (which is a generous assumption in the case of SP radiation), we can estimate the ratio of collected to total photons to be $\sin \frac{\theta^2}{2} \approx 5\%$.

These different factors, put together, can account for up to a difference of five orders of magnitude between our experimental results and the idealized theoretical model shown in Figure 4. We have also neglected other potential sources of discrepancy that are more complex to describe quantitatively: fabrication defects, surface roughness, carbon deposition on the sample from the electron beam, grazing angle of incidence, beam divergence, etc.

Supplementary References

[1] H. Rong, R. Jones, A. Liu, O. Cohen, D. Hak, A. Fang, and M. Paniccia, "A continuous-wave Raman silicon laser," *Nature*, vol. 433, pp. 725–728, feb 2005.

[2] S. Deshpande, I. Bhattacharya, G. Malheiros-Silveira, K. W. Ng, F. Schuster, W. Mantei, K. Cook, and C. Chang-Hasnain, "Ultracompact Position-Controlled InP Nanopillar LEDs on Silicon with Bright Electroluminescence at Telecommunication Wavelengths," *ACS Photonics*, vol. 4, pp. 695–702, mar 2017.

[3] S. Cueff, J. Manel Ramírez, J. A. Kurvits, Y. Berencén, R. Zia, B. Garrido, R. Rizk, and C. Labbé, "Electroluminescence efficiencies of erbium in silicon-based hosts," *Applied Physics Letters*, vol. 103, p. 191109, nov 2013.

[4] W. L. Ng, M. A. Lourenço, R. M. Gwilliam, S. Ledain, G. Shao, and K. P. Homewood, "An efficient room-temperature silicon-based light-emitting diode," *Nature*, vol. 410, pp. 192–194, mar 2001.

[5] M. A. Green, J. Zhao, A. Wang, P. J. Reece, and M. Gal, "Efficient silicon light-emitting diodes," *Nature*, vol. 412, pp. 805–808, aug 2001.

[6] L. Lumerical Solutions Inc., "http://www.lumerical.com/tcad-products/fdtd/."

[7] F. J. García De Abajo, "Optical excitations in electron microscopy," *Reviews of Modern Physics*, vol. 82, pp. 209–275, feb 2010.

[8] J. B. Pendry and L. Martín-Moreno, "Energy loss by charged particles in complex media," *Physical Review B*, vol. 50, pp. 5062–5073, aug 1994.

[9] A. P. Potylitsyn, M. I. Ryazanov, M. N. Strikhanov, and A. A. Tishchenko, "Diffraction Radiation from Relativistic Particles," *Springer Tracts in Modern Physics*, vol. 239, 2010.

[10] Y. Yang, A. Massuda, C. Roques-Carmes, S. E. Kooi, T. Christensen, S. G. Johnson, J. D. Joannopoulos, O. D. Miller, I. Kaminer, and M. Soljačić, "Maximal spontaneous photon emission and energy loss from free electrons," *Nature Physics*, p. 1, 2018.

[11] S. J. Smith and E. M. Purcell, "Visible light from localized surface charges moving across a grating," *Physical Review*, vol. 92, no. 4, p. 1069, 1953.

[12] V. Ginzburg and I. Frank, "Radiation of a uniformly moving electron due to its transition from one medium into another," *Zh.Eksp.Teor.Fiz.*, vol. 9, pp. 353–362, 1945.

[13] B. J. M. Brenny, T. Coenen, and A. Polman, "Quantifying coherent and incoherent cathodoluminescence in semiconductors and metals," *Journal of Applied Physics*, vol. 115, p. 244307, jun 2014.

[14] D. Liang and J. E. Bowers, "Recent progress in lasers on silicon," *Nature Photonics*, vol. 4, pp. 511–517, aug 2010.

[15] J. Goldstein, D. Newbury, D. Joy, C. Lyman, P. Echlin, E. Lifshin, L. Sawyer, and J. Michael, "Scanning Electron Microscopy and X-ray Microanalysis," in *Scanning Electron Microscopy*, 2003.

[16] R. A. Levy, M. L. Green, and P. K. Gallagher, "Characterization of LPCVD Aluminum for VLSI Processing," *Journal of The Electrochemical Society*, vol. 131, p. 2175, sep 1984.

[17] D. Temple, "Recent progress in field emitter array development for high performance applications," *Materials Science and Engineering: R: Reports*, vol. 24, pp. 185–239, jan 1999.

[18] S. A. Guerrera and A. I. Akinwande, "Silicon Field Emitter Arrays With Current Densities Exceeding 100 A/cm2 at Gate Voltages Below 75 V," *IEEE Electron Device Letters*, vol. 37, pp. 96–99, jan 2016.

[19] S. A. Guerrera and A. I. Akinwande, "Nanofabrication of arrays of silicon field emitters with vertical silicon nanowire current limiters and self-aligned gates," 2016.

[20] B. W. J. McNeil and N. R. Thompson, "X-ray free-electron lasers," *Nature Photonics*, vol. 4, pp. 814–821, dec 2010.

[21] C.-M. Tang, T. A. Swyden, K. A. Thomason, L. N. Yadon, D. Temple, C. A. Ball, W. D. Palmer, J. E. Mancusi, D. Vellenga, and G. E. McGuire, "Emission measurements and simulation of silicon field-emitter arrays with linear planar lenses," *Journal of Vacuum Science & Technology B: Microelectronics and Nanometer Structures*, vol. 14, p. 3455, nov 1996.

[22] S. Tsujino, P. Helfenstein, E. Kirk, T. Vogel, C. Escher, and H.-W. Fink, "Field-Emission Characteristics of Molded Molybdenum Nanotip Arrays With Stacked Collimation Gate Electrodes," *IEEE Electron Device Letters*, vol. 31, pp. 1059–1061, sep 2010.

[23] A. Mustonen, V. Guzenko, C. Spreu, T. Feurer, and S. Tsujino, "High-density metallic nano-emitter arrays and their field emission characteristics," *Nanotechnology*, vol. 25, p. 085203, feb 2014.

[24] H. Shim, L. Fan, S. G. Johnson, and O. D. Miller, "Fundamental Limits to Near-Field Optical Response over Any Bandwidth," *Physical Review X*, 2019.

[25] N. Izhaky, M. T. Morse, S. Koehl, O. Cohen, D. Rubin, A. Barkai, G. Sarid, R. Cohen, and M. J. Paniccia, "Development of CMOS-Compatible Integrated Silicon Photonics Devices," *IEEE Journal of Selected Topics in Quantum Electronics*, vol. 12, pp. 1688–1698, nov 2006.

[26] D. Lemoine, "Vacuum Packaging at the Wafer Level for the Monolithic Integration of MEMS and CMOS," no. June, 2009.

[27] M. EL-GAMAL, D. Lemoine, P.-V. Cicek, and F. NABKI, "Low temperature wafer level processing

for MEMS devices," mar 2009.

[28] M. Behnam, G. V. Kaigala, M. Khorasani, P. Marshall, C. J. Backhouse, and D. G. Elliott, "An integrated CMOS high voltage supply for lab-on-a-chip systems," *Lab on a Chip*, vol. 8, pp. 1524–1529, aug 2008.

[29] M. Behnam, G. Kaigala, M. Khorasani, S. Martel, D. Elliott, and C. Backhouse, "Integrated circuit-based instrumentation for microchip capillary electrophoresis," *IET Nanobiotechnology*, vol. 4, no. 3, p. 91, 2010.

[30] C. D. García, Y. Liu, P. Anderson, and C. S. Henry, "Versatile 3-channel high-voltage power supply for microchip capillary electrophoresis," *Lab Chip*, vol. 3, pp. 324–328, nov 2003.

[31] L. Reimer, "Scanning Electron Microscopy: Physics of Image Formation and Microanalysis, Second Edition," *Measurement Science and Technology*, 2000.

[32] J. Goldstein, D. E. Newbury, D. C. Joy, C. E. Lyman, P. Echlin, E. Lifshin, L. Sawyer, and J. R. Michael, *Scanning Electron Microscopy and X-ray Microanalysis*. 2003.
